# Living with autoinflammatory diseases: identifying unmet needs of children, adolescents and adults

**DOI:** 10.1186/s12969-018-0300-7

**Published:** 2018-12-20

**Authors:** Gabriele Erbis, Kirstin Schmidt, Sandra Hansmann, Tetiana Sergiichuk, Christine Michler, Jasmin B. Kuemmerle-Deschner, Susanne M. Benseler

**Affiliations:** 1 Rheumatology, Department of Pediatrics and autoinflammation reference center Tuebingen, University Children’s Hospital Tuebingen, Tuebingen, Germany; 2 Institute of Education, University of Tuebingen, Tuebingen, Germany; 3 Division of Oncological Surgery, Neurosurgery, Urology Gynaecologic Surgery District Clinics Reutlingen, Reutlingen, Germany; 4 Rheumatology, Department of Paediatrics, Alberta Children’s Hospital, Cumming School of Medicine, University of Calgary, Shaganappi Trail NW, Calgary, Alberta T3B 6A8 Canada

**Keywords:** Autoinflammatory disease, Rare disease, Unmet needs, Health-related quality of life, Psychosocial impact, Intervention, Health communication, School performance, School bullying, Participation

## Abstract

**Background:**

Autoinflammatory diseases (AIDs) illnesses of the innate immunity resulting in clinical signs and symptoms of systemic inflammation and loss of organ functions. While pathophysiological mechanisms are heavily studied and increasingly well understood, psychosocial needs are much less explored. The disease impact on the everyday life of patients including school and work is poorly studied. The purpose of the study was to identify the spectrum of unmet needs of children, adolescents and adults living with autoinflammatory disease and their families, to define key unmet needs and strategies and to develop and evaluate a pilot intervention addressing the unmet need “school”.

**Methods:**

A single-center, mixed-method study of AID patients and their families was conducted. Consecutive patients ages ≥4 years and their families were included. Expert consulting, focus groups and questionnaires explored the patient perspective of “unmet needs in AID”. Quantitative and qualitative content analyses were performed and informed the development of a framework of unmet needs. A targeted pilot multimodular intervention for the unmet need “school” was developed and tested. Health-related Quality of Life (HRQoL) was evaluated using DISABKIDS-questionnaires and psychosocial impact evaluations.

**Results:**

The study included 83 patients and their families. These were 14 children, 9 adolescents and 25 adults with AID and 35 family members; patients’ median age was 19 years (5–78). Expert consultations: 110 AID patients with 320 visits/year; 99 (90%) were children and adolescents. 78 patients and family members (94%) participated in 10 groups. Qualitative content analysis delineated 9 domains of unmet needs, the most relevant being school, health care system and public institutions. The pilot intervention“school” included 18 participants; median age was 9 years (7–16). HRQoL improved with the intervention including “understanding” by 53%, however improvement was not sustained over time.

**Conclusion:**

Unmet needs of AID patients and families affect all areas of life. Accessible networks increasing knowledge and empowering patients, strategies supporting academic and workplace environments to ensure successful participation and integrated concepts addressing psychosocial needs are urgently needed.

## Background

Autoinflammatory diseases (AIDs) illnesses of the innate immune system resulting in clinical signs and symptoms of systemic inflammation including fevers and severe fatigue in addition to loss of function in multiple organ systems [1, 2]. Genomic discovery and availability of genetic testing has enabled and expedited the diagnostic evaluation and decreased the delay to diagnosis [3]. It also frequently resulted in deciphering extensive autoinflammation pedigrees following the diagnosis of an index patient [4]. Understanding the biology of autoinflammation enabled physicians to rapidly select effective therapies to target the affected inflammasome and its key mediators such as Interleukin-1 (IL-1) and control disease activity [5]. These therapies are highly effective, yet expensive and required long-term use to prevent organ damage such as hearing loss or renal failure [1].

The World Health Organization defined Health Related Quality of Life (HRQoL) as the person’s perceptions of the position in life in the context of their culture and value systems in which they live and in relation to their goals, expectations, standards and concerns [6–8]. Impaired HRQoL was recently found to be a hallmark of AIDs as the diseases not only severely impact physical abilities, but also social and psychological functioning including work productivity and educational attainment [9–13]. Most often, AIDs were shown to affect patients’ and their families, personal relationships, school situation, employment or social activities [9]. In addition, mood conditions were reported to be associated with AIDs such as Familiar Mediterranean Fever (FMF) adding to the overall emotional disease burden [14].

While physical signs and symptoms are often effectively controlled by cytokine blockers such as IL-1 inhibition or other therapies, psychosocial needs are much less explored and often not sufficiently addressed, even in treated patients such as those with Hyper Immunoglobulin-D syndrome [13]. In fact, the impact of AIDs on the activities of daily life of patients such as school and work is poorly studied. The unmet needs of children and adults with AIDs remain to be clearly defined and strategies to address these unmet needs remain to be developed.

The aims of the study were 1) to identify the spectrum of unmet needs of children, adolescents and adults living with autoinflammatory diseases and their families, 2) to define key unmet needs and core strategies to address these and 3) to develop and evaluate a pilot intervention which addresses the unmet need “school”.

## Methods

The study was a mixed-method approach including expert consulting, scoping review, focus groups and questionnaires evaluating the patient perspective of the “unmet needs in AID”. Subsequently, a multimodular pilot intervention for the unmet need “school” was developed and evaluated using DISABKIDS-questionnaires and psychosocial impact evaluations.

Patients with autoinflammatory diseases and their families cared for at the autoinflammation reference center Tuebingen (arcT) were invited to two consecutive annual “arcT patient information days” and asked to participate in the study. The study timeframe was February 2015 to December 2016. The study excluded children under the age of 4.

### Expert consulting and scoping review

An interprofessional expert team consisting of health care providers, teachers and social workers with expertise in educational science, psychosocial care and family therapy developed a framework for the evaluation of unmet needs in AIDs for the different areas of life. The participating expert in psychosocial care and support (GE) contributed her expertise of 15 years of coaching of children and families with AIDs. She specifically contributed data documenting the significant social and emotional burden of AID. A scoping review was conducted to identify published unmet needs in AID and other rare diseases and review the methodological approaches to evaluate disease burden and impact on HRQoL when living with rare diseases.

### Focus groups

The annual “arcT patient information day” provides updates of important medical and psychosocial information for patients and families. It also offers the opportunity to exchange experiences and to explore the unmet needs of people living with AIDs and their families. All participants were invited to join the focus groups. At the first arcT patient information day AID patients were grouped primarily by age into groups of children, adolescents and adult patients in addition to family members including partners and parents. The task was to identify key domains of unmet needs in AID. The subsequent year, domain specific focus groups further explored each area of unmet needs. A total of 10 focus groups were established. Each group had a defined number of participants and 2 moderators. Semistructured interview questions guided the focus group conduct. All interviews were recorded and transcribed. Qualitative analysis was performed according to the Mayrings Qualitative Content Analysis [15] A category system was created consisting of deductive and inductive categories (domains), subcategories (subdomains) and examples using participants’ citations. Frequencies of the categories were quantified. Passages in the transcripts were matched with the corresponding category or subcategory using the Qualitative Data Analysis (QDA) Miner software (Version 3.2, Provalis Research).

### Patient questionnaire “Unmet needs in AID”

A patient questionnaire “Unmet needs in AID” was developed to further quantify and weigh the unmet needs and their impact. The domains of the questionnaire included path and time to diagnose, knowledge about AIDs in health care providers, school and work related challenges such as missing school/work days, knowledge gaps about social rights and strategies to cope with the disease.

### Pilot intervention targeting unmet need

Information derived from psychosocial consultations, focus groups and quantification of the key domains as well as the impact assessment was reviewed. Based on these data the first pilot intervention targeting the core unmet need in AID “school” was defined.

### Pilot intervention

A multimodular, tailored pilot intervention for “school” was developed and implemented in an inception cohort of children and adolescents with AIDs. A comprehensive strategy was developed and integrated written information about AID, telephone conferences and multiprofessional (clinic teachers and social workers) school visits. The aims of the pilot intervention were defined by the results of the psychosocial consultations, scoping review and the information derived from focus groups. Accompanying psychosocial impact evaluations were offered by a social worker to analyze the school situation.

### Outcome

The impact of the pilot intervention on HRQoL in AID patients was evaluated using the DISABKIDS-questionnaire, a validated, generic instrument for children and adolescents with chronic disease and their parents [16]. The arthritis module was selected since there was no specific module for AID. Correspondingly for AID and arthritis the domain “understanding” captures the reactions from teachers, parents and friends in terms of understanding of disease related symptoms. Secondary outcomes included the transformed score in the domain “impact”, which encompasses the individual’s disease burden and the total transformed score for HRQoL. Patients and parents completed the DISABKIDS-questionnaire monthly for two month prior and post intervention.

### Analysis

Descriptive statistics were used to compute means, standard deviation, medians, ranges and frequencies. Comparative analysis utilized parametric and non-parametric tests as appropriate.

## Results

### Patients

The study cohort consisted of 83 children and adults including AID patients and their family members. A total of 48 (58%) were AID patients including 14 children (29%) ages < 14 years, 9 adolescents (19%) ages 14–21 years and 25 adults age >  21 years (52%). The median age of the patients was 19 years (range 5–78 years), 18 (38%) were female. A total of 37 patients were diagnosed with Cryopyrin-Associated Periodic Syndrome (CAPS) (77%), including 35 patients with the moderate phenotype Muckle-Wells-Syndrome (MWS) and two with the severe phenotype neonatal-onset multisystem inflammatory disease (NOMID)/chronic infantile neurologic, cutaneous and articular (CINCA) syndrome. None of the patients had the mild CAPS phenotype, familial cold autoinflammatory syndrome (FCAS). Ten patients were diagnosed with FMF (21%) and one with an unclassified AID (2%). A total of 35 family members (42%) took part in the study; these were all adults (Table 1).

**Table 1:** Demographics of study participants with autoinflammatory diseases (AID) and their family members

	Total study cohort of AID patients and family members *N* = 83
Patients with AID (%)	48 (58%)
Male: female	30:18
Median age at study in years (range)	19 (5–78)
• Children (5 < 14 years), N (%)	• 14 (29%)
• Adolescents (14–21 years), N (%)	• 9 (19%)
• Adults (> 21 years), N (%)	• 25 (52%)
AID subtypes	
• Cryopyrin-Associated Periodic Syndrome (CAPS), N (%)	• 37 (77%)
- Familial cold-associated syndrome (FCAS)	0
- Muckle-Wells-Syndrome (MWS)	35/37
- Neonatal-onset multisystem inflammatory disease (NOMID)/chronic infantile neurologic, cutaneous and articular (CINCA) syndrome	2/37
• Familial Mediterranean Fever (FMF), N (%)	• 10 (21%)
• other AIDs, N (%)	• 1 (2%)
Total AID family members, N (%)	35 (42%)
Focus groups	
Total number of focus groups	10
Total participants of focus groups (%)	78 (94%)
• AID patients, N (%)	• 45 (58%)
○ Children/adolescents N (%)	○ 19 (24%)
○ Adults, N (%)	○ 26 (33%)
• AID family members N (%)	○ 33 (42%)
“Impact and unmet needs in AID” questionnaires	
Total questionnaires completed (%)	40/43 (93%)
• Adolescent and adult AID patients	• 40
Male: female	• 21:18 (no response in one)
	Inception cohort “Pilot School Intervention in AID”
Total number of participants	18
• Children and adolescents with AID, N (%)	• 9 (50%)
• AID parents, N (%)	• 9 (50%)
Male: female	6:12
AID patients median age in years (range)	9 years (7-16)

### Expert consulting and scoping review

Both expert consulting and scoping review determined the unmet needs in specific areas of life such as family, school and work. Psychosocial consultation in arcT between 2000 and 2015 included a total of 110 AID patients with 320 visits/year; 99 (90%) were children and adolescents. Frequently family members required significant psychosocial support, too. The key theme of unmet need addressed in these consultations was “school”. Data informed the structure and conduct of the focus groups.

### Focus groups – UNMET needs

**Fig. 1:**
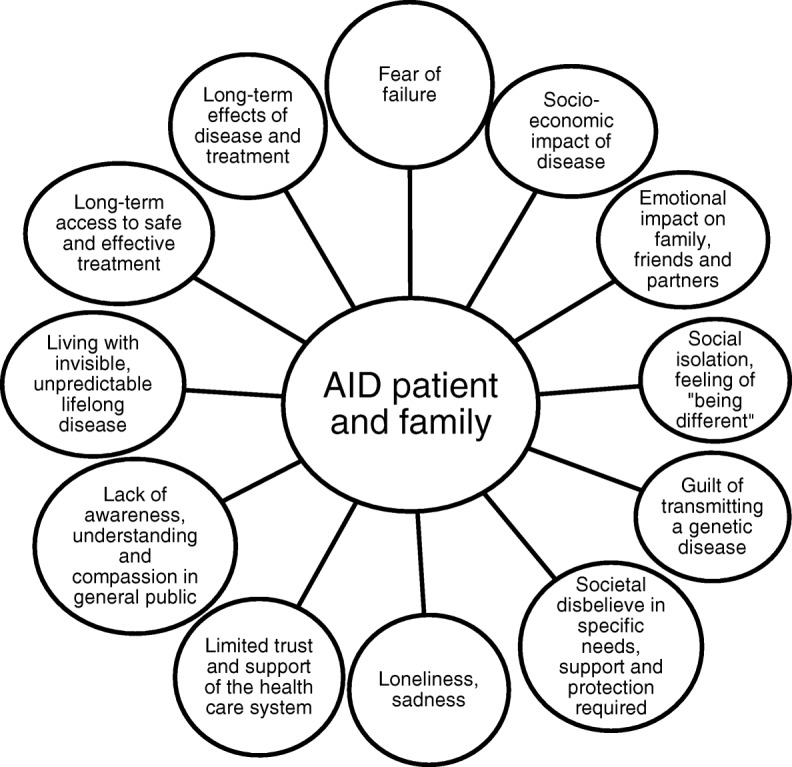
Psychosocial burden of patients and their family members with autoinflammatory diseases (AID). The figure summarizes the key themes identified in focus groups of children, adolescents and adults with AID and their family members. It visualizes the areas of emotional burden of living with AID. Each circle refers to a specific burden, affecting all areas of life

A total of 10 focus groups were established with a median of six participants. A total of 78 patients and family members participated; these were 94% of the total study cohort. Of these 45 (58%) were patients including 19 (24%) children and adolescents and 26 (33%) adults. A total of 33 (42%) were family members including parents and partners (Table 1). Qualitative content analysis delineated the perceived burden of AIDs for patients and family members (Fig. 1). Furthermore, it resulted in domains and subdomains of unmet needs based on participant’s feedback. A total of 9 domains with an average of 4 (range 3 to 8) subdomains (range 3–8) reflected the identified areas of unmet needs. The domains and subdomains are listed and supported by citations:

- School: understanding/compassion, integration and bullying, attendance, participation/performance and accommodation of health-related needs. Citation: “Children in school are often judged incorrectly, we are so tired. The teachers think they are not motivated, does not want to learn. The extreme fatigue is part of our illness” (Focus group adult patients, P9, #00:25:56–5#)
- Health care: understanding/experiences in the health care system, knowledge of health care providers in diagnosing and managing rare disease, close to home medical care provision, AID centers, health insurances, emergency and staying abroad. Citation: “I believe physicians close to home have no information about autoinflammatory diseases. In fact, they are simply not aware that these diseases exist.” (Focus group parents, P7 #00:19:07–7#)
- Public institutions: psychosocial counseling/support, administrative bodies, social services such as pensions, disabilities, life insurance and public support services. Citation: “We wanted to get a life insurance for our child when she was 4 or 5 years old. We did not get one, we were told the child will die soon anyways, therefore she will not get one.” (Focus group family members P4#00:14:28–5#)
- Work: understanding/compassion and bullying, work attendance/missing workdays, limited workplace selection and career options, job security – concerns of job loss. Citation: “ He got really sick, also mentally, the constant pressure of his employer was making him unstable and labile.” (Focus group family members, P2#00:07:39–3#)
- Friends/peers: social limitations – physical and emotional, participation/social functioning and vulnerability. Citation: “... we often have to cancel social gatherings, because our child has a disease flare […] and our friends don’t understand: why are they cancelling again? We don’t really get invited any more.” (Focus group adult patients, P2#00:17:19–4#)
- Individual living with AID: understanding/compassion, physical functioning, emotional burden, self-esteem/self-doubt, vitality, guilt, family planning/genetic risk and confidentiality/information privacy concerns. Citation: “I have to tell people, my child is sick and needs the needle because he inherited the disease from me. It was very hard to for me in the beginning.” (Focus group adult patients, P7, #01:08:13–7#)
- Knowledge: general public awareness, knowledge about rare disease and treatment options: efficacy/safety information for patients.
- Citation: “ Side effects, yes I have them, but I am not sure how to continue? Not sure the medication really works well, not sure what else I can do.” (Focus group adult patients, P1 #01:11:52–9#).
- Family: parental burden, family members understanding/compassion as well as leisure activities. Citation: “ the psychological pressure ... is much higher for parents with sick children. Everything comes with a huge time commitment.” (Focus groups adult patients, P3, #01:07:10–3)
- AID network: information, share experiences/coping, overcoming isolation and social support. Citation: “Would be great to learn how others cope with this. Perhaps they can talk to someone. How they handle living with the symptoms, the pain.” (Focus group adolescents patients, P3, #00:58:39–4#)

### Quantification of domains

**Fig. 2:**
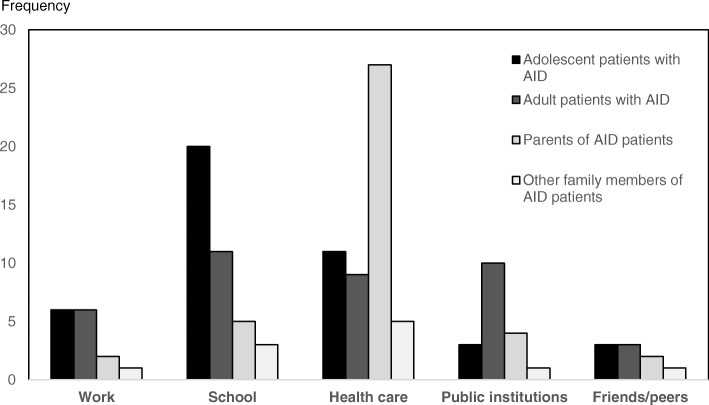
Key domains unmet needs of patients with Autoinflammatory Diseases (AID) and their family members. The frequencies of key domains of disease impact on daily living were identified in focus groups. Those frequencies were defined for the four different groups, adolescent patients with AID, adult patients with AID, parents of AID patients and other family members of AID patients. The domains health care, school and public institutions were the three most often named areas of AID disease impact

Patients and their family members’ estimates were quantified to determine the most relevant domains of unmet needs. Overall the three key domains were the health care system, school and public institutions. The first domain specified the lack of knowledge, understanding and compassion in the “health care system” including providers and insurances. The “school” domain included the lack of knowledge, understanding, compassion and bullying of teachers and other students and the patients’ challenges with performance and participation. The domain “public institutions” captured the lack of support of several public offices such as social services. While parents and other family members emphasized the deficits in the “health care system”, patients clearly determined the domain “school” as the primary unmet need in AID (Fig. 2).

### Patients’ questionnaire “Unmet needs in AID”

A total of 40/43 (93%) questionnaires were completed (Table 1). These were 21 males and 18 females (no response in one). Of these 33 (83%) were treated, most commonly with anti-IL1 therapy. Only 8 patients (20%) reported that they were diagnosed within 3 to 5 years of symptom onset, while 16 (40%) documented a time to diagnosis of 5 to 15 years and 8 (20%) more than 15 years (no response in 8). A total of 10 (25%) were single, 18 (45%) in a relationship, while 12 provided no answer. A total of 13 (33%) reported to be in school, 17 (43%) were working, while 4 (10%) were unemployed and 3 retired or on disability (no response in 3). The most commonly reported area of information deficit was the general practitioner identified by 34 (85%). Only 10 (25%) documented that they were informed about the applicable social security laws and social rights. A total of only 13 (33%) reported to have access to psychosocial support. Disease impact: 19/30 responders (63%) reported an average of 3 days of missing school or work per month; 6/30 (20%) even documented an average of 5 days/month (Table 2).

**Table 2:** Unmet needs in autoinflammatory diseases (AIDs): Results from adolescent and adult patients’ questionnaire

Questionnaires completed	40/43 (93%)
Male: female	21:18 (no response in one)
Time to diagnose from symptom onset	
• Within one year	• 0
• 1–2 years	• 0
• 2–3 years	• 0
• 3–5 years	• 8 (20%)
• 5–15 years	• 16 (40%)
• > 15 years	• 8 (20%)
• No response	• 8 (20%)
Treatment with anti-IL1 therapy	33 (83%)
Socioeconomic data	40
• Single	• 10 (25%)
• In a relationship	• 18 (45%)
• No answer	• 12 (30%)
• In school	• 13 (33%)
• At work	• 17 (43%)
• Unemployed	• 4 (10%)
• Retired/on disability	• 3 (8%)
• No response	• 3 (8%)
Identified areas of key unmet needs and information deficits	
• Knowledge deficit of general practitioner	• 34 (85%)
• Access to information about the applicable social security laws and social rights	• 10 (25%)
• Access to psychosocial support	• 13 (33%)
Impact of AID in the group of patients, who are in school or employed	30/40 (75%)
Average missing days at school/work	
• Three days per month	• 19 (63%)
• Five days per month	• 6 (20%)
• Seven days per month	• 3 (10%)
• > 2 weeks per month	• 1 (3%)
• No response	• 1 (3%)

### Unmet needs

The comprehensive mixed-method study including expert consulting, scoping review, focus groups and questionnaires identified and quantified the “unmet needs in AID”. From a total of nine identified domains, children, adolescents and their family members highlighted school, health care system and public institutions as the key areas of unmet needs. The challenges for each domain were detailed and the impact of living with an autoinflammatory disease was documented.

### Pilot intervention targeting key unmet need

The first intervention was co-produced with patients and parents targeting the key unmet need “school”. The inception cohort consisted of 26 participants including 13 AID patients and one of their parents. A total of 18 completed the study, the remaining 8 dropped out prior to study completion. The median age of the patients was 9 years (range: 7–16 years) (Table 1).

### Pilot intervention for core unmet need “school”

Based on the results of the focus groups the aims of the pilot intervention were to inform teachers and other students about AIDs, the disease impact on school and daily living focusing on performance, participation and accommodation of health related needs hypothesizing that provision of information improves teachers’ and students’ understanding and compassion. Strategies were provided to teachers and classmates to identify individual disease related needs, compensate for disadvantages of students suffering from AID and to empower patients. The multimodulare approach included four steps:

The multimodulare approach included four steps:

- Consultation: Psychosocial consultations including patients, care givers and social workers identified individual problems at school including lack of information/knowledge and challenges in interactions/behaviors.
- Information letter: Generic information letters co-produced by families, social workers and clinic teachers summarized AID specific symptoms and disease impact on performance and participation at school for teachers.
- School consultation: Joint consultations of the AID care team and the school explored the school-related challenges and developed potential strategies to provide information and improve interactions and behaviors.
- School visit: An interactive, focussed school visit is co-designed and conducted by the patient, family, AID care team and the school aiming to implement effective and sensible strategies for information sharing, support and optimal interactions.

Accompanying psychosocial impact evaluations revealed that missing school days (> 1 week/month) were documented in 7 out of 9 patients (78%). AID-related cognitive deficits were identified by 8 (89%), social exclusion by 7 (78%) and suicidal ideation by 3 patients (33%). Additional themes included lack of understanding and compassion of classmates and teachers, bullying, aggressive behavior and truancy.

### Outcome

#### Primary outcome

**Fig. 3:**
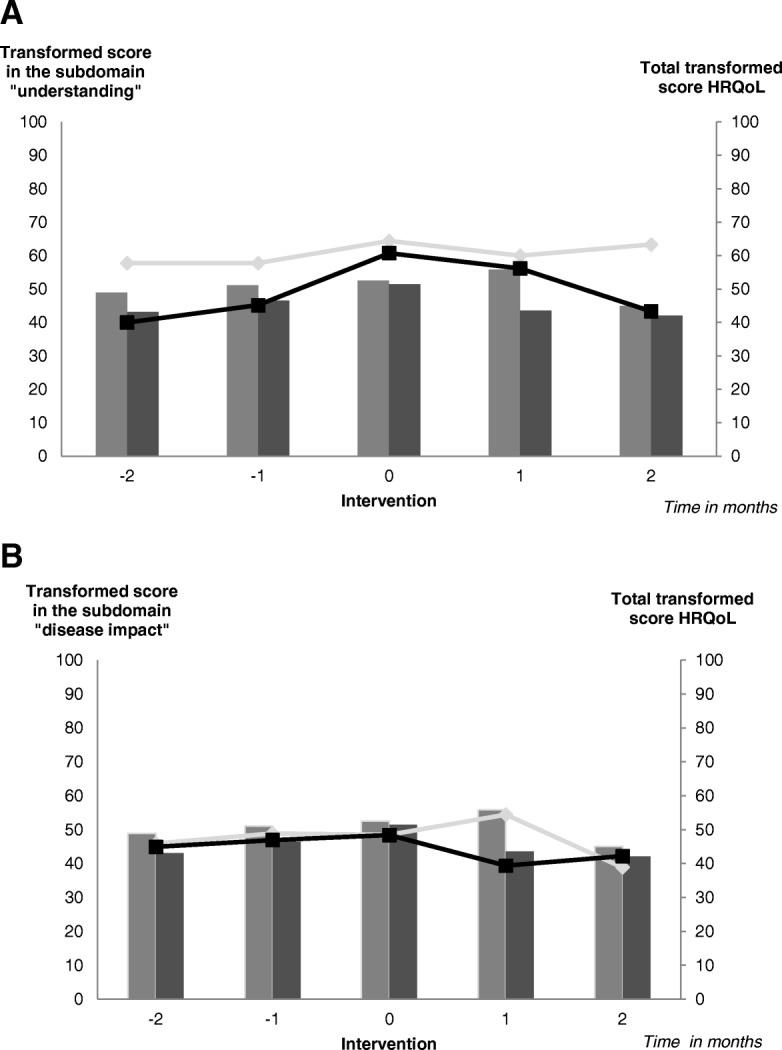
Effect of the pilot school intervention on Health Related Quality of Life in autoinflammatory diseases. The effect of the pilot school intervention on the transformed score in the domain understanding, disease impact and the total transformed score Health Related Quality of Life (HRQoL) was determined using the DISABKIDS instrument in children with autoinflammatory diseases and their parents. Children/Adolescents with AID (light grey) and their parents (dark grey) assessed the effect of the intervention on the transformed score in the domain understanding (lines **a**), the domain impact disease (lines **b**) and the total transformed score HRQoL (bars **a** and **b**). The effect of the intervention was evaluated by the DISABKIDS-questionnaire. According to the domain understanding (**a**), impact understanding (**b**) and the total score HRQoL (**a** and **b**) the intervention has a positive, yet not sustained effect on the HRQoL of patients with AID

Prior to the intervention the mean “understanding” score of the DISABKIDS questionnaire was 58/100 (SD ±17.7) in children and 40/100 (SD ±24.5) in parents. At time of school intervention, the mean score of children increased to 64 (SD ±16.7) and parents to 61 (SD ±13.1), the latter representing an increase of 53%. Two month post intervention the score of children decreased to 63 (SD ±4.7) and to 43 (SD ±11.5, Δ -29.5%) in adults (Fig. 3a).

#### Secondary outcomes

The baseline “impact” score of children and adults prior to intervention was similar, 46 in children (SD ±8.5) and 45 in parents (SD ±12.3). At time of intervention, the scores increased slightly in both groups, in children to 49 (SD ±16.5) and in parents to 48 (SD ±15.6). The post intervention score in children further increased to 54 (SD ±15.3) at one month, however subsequently dropped below baseline at 2 months (39) (Fig. 3b).

The baseline HRQoL total score was higher in children at 49 (SD ±7) than adults 43 (SD ±10.5). The score improved in both groups, in children to 53 (SD ±11.5) and in adults to 51 (SD ±11.7). The post intervention scores were dramatically lower in both groups, in children 45 (SD 0), in parents 42 (SD ±1.6) (Fig. 3a and b).

## Discussion

This was the first study to explore the unmet needs of children, adolescents and adults living with AIDs and their family members using a mixed-methods approach. Key unmet needs were identified in focus groups, enriched in individual psychosocial consultation, categorized in domains and subdomains and weighted in questionnaires. Thereby “school”, “health care” and “public institutions” were identified as the most important domains of unmet needs. A multimodular pilot intervention “school” was developed aiming to improve the HRQoL of students living with AIDs. The evaluation using the validated DISABKIDS-questionnaires demonstrated a positive yet not sustained effect.

The study’s multimodular approach clearly demonstrated “school” being the key unmet need identified by 90% of children and adolescents with AID resulting in significant psychosocial burden documented in more than 320 annual psychosocial counseling visits. Focus groups of 78 children, adolescents and adults echoed this unmet need and “missing school” was the central theme patients documented in questionnaires. An average of 3 days of missing school per month was reported by 63% of patients, a mean of 5 days by 20%. These results are similar to data published in a recent French cohort study of children with CAPS: Kone-Paut et al. reported a disease-related school absence of 0.9 days/week [17]. The school-related burden of AID was previously documented by Stych et al.; in this survey study 80% of FCAS patients revealed their symptoms becoming burdensome by school age. To cope with their disease and to avoid disease flares, FCAS patients reported limiting participation in school, family, and other social activities [18]. The critical importance of “school” for children and families living with AID mandates a proactive approach towards strategies to decrease this burden.

“Health care” was the second most important area of unmet needs identified by AID patients and their families. A lack of knowledge of general practitioners associated with a significant deficit in information provided to patients was documented in 85% of AID patients. This was echoed by patients and their family members in focus groups emphasizing the perceived lack of understanding and compassion of health care providers towards them. The same theme was documented in large studies of rare diseases patients: 56% of 837 patients and family members with rare diseases rated the knowledge of their primary care physicians as only “fair” or even “poor”. Diagnoses were made primarily by specialists (44%). Engel et al. reported that many primary care physicians agreed (15%) or were neutral (24%) about the statement “I can’t get involved with the diagnosis of a rare disease, there are just too many of them for me to be aware of” [19]. This perceived lack of knowledge caring for children and adults with rare diseases was also clearly documented by Budych et al. who conducted interviews with 107 patients living with six different rare diseases [20]. The authors emphasized that patients with rare diseases were uniquely forced to become knowledgeable about their disease and treatments. The study highlighted the resulting shift in the traditional roles in medical encounters and the altered patient–physician relationships [20]. These results support the urgent need for information sharing networks empowering patients with rare diseases such as AIDs.

Addressing the core unmet need “school” with a multimodular pilot intervention demonstrated a positive, yet not sustained effect on HRQoL. The pilot school intervention resulted in a significant increase of the perceived parental perception of “understanding” of the social environment (53%). The child’s report of the “understanding” component of HRQoL also improved immediately following the intervention, however to a far lesser extent (10%). Two month post intervention, the parental perception of “understanding” dropped by 30% close to baseline pre-intervention revealing the lack of sustained effectiveness. In contrast, published school interventions for childhood epilepsy demonstrated a sustained effect at the six month follow-up [21]. Bracbcova et al. utilized an information video (*N* = 89) and a personal story (*N* = 93) to improve disease-related knowledge and negative perceptions of students towards their peers. The authors demonstrated a significantly sustained impact of storytelling on the stigma of epilepsy as measured by the Stigma Scale of Epilepsy (improved by 25%) and improved overall knowledge (by 55%) [21]. The difference in the results between pilot school interventions in AID and epilepsy may be explained by multiple factors including the prior public knowledge of epilepsy being a more common disease, the choice of outcome measure - generic HRQoL versus focused measures, and the difference in who was asked - patients versus fellow students. While reducing the perceived stigma and improving knowledge of peers are important aspects, the impact of these interventions on well-being and HRQoL of students living with rare diseases remain to be explored.

In our study the significant psychosocial burden of AID was specifically detailed in the accompanying psychosocial impact evaluations of patients participating in the pilot school intervention. Students were found to have more than one week of missing school each month in 78%, cognitive deficits in 89%, social exclusion and marginalization in 78% and suicidal ideation in 33%. A recent systematic meta-review by Lum et al. reported similar results for children and adolescents living with different chronic illnesses [22]. Chronic illness was associated with significantly higher rates of missed school days compared to healthy students. Grieve et al. reported that children with cystic fibrosis were absent from school an average of 23.6 days per year resulting in an attendance rate of only 86.9%; in comparison, North American national attendance rates for all high school students for school years 2006–2009 ranged from 92.7 to 93.3% [23]. Impaired relationships with teachers and fellow students including bullying and other negative behaviors displayed by teachers and peers were identified [22]. Lum et al. reported that greater support in school was associated with better educational outcomes, school experiences and fewer absenteeism [22].

There were several limitations to the study. It had a small sample size in particular for the pilot school intervention. However, AIDs are rare diseases occurring in less than 1/million. The total number of 83 study participants represented one of the largest cohorts of patients and family members with AID studied to date. The school intervention should be strictly viewed as a pilot and should be replicated in a bigger cohort of patients. Suitable outcome measures for interventions in AIDs are limited. For the present study the DISABKIDS-questionnaires were considered to be the most relevant in particular the domains “understanding” and “impact”. The study included primarily patients with CAPS and FMF potentially limiting the generalizability to other AIDs. However the challenges of people living with AIDs are very similar across autoinflammatory diseases. Unmet needs and psychosocial burden is related to the countries’ socioeconomic status especially access to health care and expensive medications. In developed countries “school” is a critically important unmet need as demonstrated in this study.

## Conclusions

The unmet needs of people living with rare diseases such as AIDs are complex and affect all areas of life. Consistently patients and families documented their struggle with the rarity and invisibility of the diseases resulting in a significant lack of understanding and compassion of their social environment. This included the extended family, friends, health care providers, school and work further increasing the burden of their illnesses. Researchers and health care providers closely partnered with patients have to create comprehensive concepts to decrease the individual burden of rare diseases such as AIDs. Accessible networks to increase knowledge and empower patients, transferrable strategies to support the academic and workplace environments to ensure successful participation and comprehensive integrated concepts to address the psychosocial needs are urgently required.

## Abbreviations

AID: Autoinflammatory disease
arcT: Autoinflammation reference center Tuebingen
CAPS: Cryopyrin-associated periodic syndrome
FCAS: Familial cold auto-inflammatory syndrome
FMF: Familiar Mediterranean Fever
HRQoL: Health-Related Quality of Life
IL-1: Interleukin-1
MWS: Muckle-Wells-Syndrome
NOMID/CINCA: Neonatal-onset multisystem inflammatory disease/chronic infantile neurologic, cutaneous and articular syndrome
